# Nanoscale Chemical Surface Analyses of Recycled Powder for Direct Metal Powder Bed Fusion Ti-6Al-4V Root Analog Dental Implant: An X-ray Photoelectron Spectroscopy Study

**DOI:** 10.3390/bioengineering10030379

**Published:** 2023-03-20

**Authors:** Anastasia Matsko, Nader Shaker, Ana Carla B. C. J. Fernandes, Asmaa Haimeur, Rodrigo França

**Affiliations:** 1 Biomedical Engineering Program, Faculty of Engineering University of Manitoba, Winnipeg, MB R3T 2N2, Canada; 2 Department of Restorative Dentistry, College of Dentistry, University of Manitoba, Winnipeg, MB R3E 0W2, Canada; 3 Department of Oral Biology, College of Dentistry, University of Manitoba, Winnipeg, MB R3E 0W2, Canada

**Keywords:** laser sintering, Ti-6Al-4V, reused powder, titanium implants, additive manufacturing, implant surface composition

## Abstract

Over the past couple of decades, additive manufacturing and the use of root-analogue-printed titanium dental implants have been developed. Not all powder particles are sintered into the final product during the additive manufacturing process. Reuse of the remaining powder could reduce the overall implant manufacturing cost. However, Ti-6Al-4V powder particles are affected by heat, mechanical factors, and oxidization during the powder bed fusion manufacturing process. Degradation of the powder may harm the final surface composition and decrease the biocompatibility and survival of the implant. The uncertainty of the recycled powder properties prevents implant fabrication facilities from reusing the powder. This study investigates the chemical composition of controlled, clean, and recycled titanium alloy powder and root-analogue implants (RAI) manufactured from these powders at three different depths. The change in titanium’s quantity, oxidization state, and chemical composition in powder and RAI implants have been demonstrated and analyzed. While not identical, the surface chemical composition of the recycled powder implant and the implant manufactured from unused powder are similar. The results also indicate the presence of TiO_2_ on all surfaces. Many studies confirmed that titanium dioxide on the implant’s surface correlates with better osteointegration, reduced bacterial infection, and increased corrosion resistance. Considering economic and environmental aspects, surface chemical composition comparison of clean and reused powder is crucial for the future manufacturing of cost-effective and biocompatible implants.

## 1. Introduction

During the past 40 years, the Brånemark conventional osseointegrated titanium dental implants have demonstrated excellent clinical performance. As a result, the market for this kind of medical procedure is also flourishing. In the USA, more than 5 million dental implants are placed annually, and it is estimated that more than 100 million American patients need at least one implant. Unfortunately, these conventional dental implants cannot be indicated for all clinical cases. For example, there are situations where the residual amount of alveolar bone is not enough to bear the length of the conventional screw. Another situation could involve the lack of space between two teeth preventing the placement even of a dental implant with a narrow diameter.

Ti-6Al-4V alloy is a well-known material for many biomedical applications due to its excellent mechanical properties, good corrosion resistance, biocompatibility, and light-specific weight [1,2,3]. Ti-6Al-4V is a double-phase (α + β) titanium alloy. The alignment of the atoms of titanium in the solid state is in either a hexagonal close-packed crystalline structure, called the alpha (α) phase, or a body-centred cubic structure, called the beta (β) phase. Oxidation depends on 3D processing conditions and on number of printing cycles. It affects microstructure and phase distribution in Ti-Al-4V alloy. Phase constitution is also found to be closely related to the corrosion resistance [4,5].

Titanium materials have been recently introduced in a powder form that allows additive manufacturing of easily customized products according to specific customer requirements [6]. The powder bed fusion process (PBF) or direct metal laser sintering has allowed the fabrication of such custom implants with a favourable combination of mechanical and biological properties. For example, a custom-made root-analog dental implant (RAI) has been presented as an encouraging way to repair clinical cases in which conventional conic dental implants are not indicated [7,8,9,10]. A complex geometry structure could be built using the PBF process. During the build, the material is added layer by layer via directing a high-power laser beam at a bed of titanium (Ti-6Al-4V) microparticles (25–45 μm), which fuses the particles according to a powerful laser beam controlled by a computer-aided design (CAD) file [10,11]. While conventional conic implants are machine milled and only available in standard sizes and diameters (narrow, medium, large), PBF RAI would have the advantage of allowing the fabrication of dental implants with customized sizes and diameters. This feature would permit immediate placement of dental implants after tooth extraction, avoiding a second surgery and reducing the healing time [12].

However, technical questions are still a barrier to the broad use of RAI. One of them is the fact that not all powder particles melt and solidify during the laser melting process. Since titanium powder’s cost significantly affects the cost of parts built by melting [13], recycling this metal powder has significant economic benefits to AM users [14]. Although reused powder provides cost-lowering benefits, powder degradation during additive manufacturing is consequential. Heating, mechanical, and oxidation factors impact powder properties [5]. It is well known that implant chemical composition may influence osteointegration and, thus, is vital for implant survival [15,16,17]. The biological interaction between the implant alloy and the bone will vary depending on the percent composition of the alloy components. For example, increasing the content of Al and V in the surface composition has increased the adhesion of an osteoblast-like cell line through fibronectin-promoted adhesion [18].

Meanwhile, it is known that Al and V are toxic elements. Li, X.C., et al. mentioned that the conventional Ti-6Al-4V alloy has increased toxicity compared to CP Ti powder due to the absence of Al and V in CP Ti powder [19]. Additively manufactured items, especially implants, require post-treatment. Some of the treatment is performed to reduce the residual stress [16,20,21]. Bioactivation modification heat treatment with alkaline has also been reported as one of the post-processing steps [22]. When subjected to chemical coatings or heat treatment, Ti bonds to living bone through an apatite layer formed on its surface. The layer modulates cell adhesion, migration, proliferation, and differentiation with consequent bone formation [23]. The corrosion resistance of Ti-6Al-4V alloy subjected to the laser sintering process is another question to address. It is known that alloy corrosion resistance could be improved by adding the Cu element [24]. Some researchers reported the corrosion resistance of SLM Ti-6Al-4V was noticed to be higher due to the presence of relatively higher β content in comparison to the wrought Ti-6Al-4V [25], although the failure of titanium alloys in medical implants is known to be induced by pitting and crevice corrosion [3]. Further surface modification of dental implants plays a significant role in obtaining more bio-functional materials.

Biomaterials communicate with the body through their interfaces. Although physical properties such as elastic modulus, hardness, and toughness strength are essential to bear mechanical stresses, from the biocompatibility standpoint, only the surface matters [26,27,28]. Especially for dental implants, the surface chemical composition plays a significant role in the overall success as it is liable to impact cell response [15,29,30,31]. Over the past decade, the chemical and mechanical properties of 3D-printed titanium objects [30,32,33] and titanium powders [20,34] have been broadly studied. However, a comparison of elemental composition between the first-time-used powder and PBF-processed particles is absent in the literature. The main aim of this study was to characterize the nano-level surface of dental root-analog implant samples fabricated with unspoiled and reprocessed powders. Using an X-ray photoelectron spectroscopy (XPS) tool, the chemical composition of the RAIs at three different depths (0, 10, and 100 nm) was assessed and compared with the control powder and recycled powder. Moreover, high-resolution XPS spectra were used to differentiate the oxidation state of the major elements Ti, O, Al, and V in the four experimental conditions tested in this study.

## 2. Materials and Methods

Titanium alloy powder (EOS Titanium Ti64, E.O.S. GmbH, Krailling, Germany) was used in this experiment under two different conditions: one from the manufacturer directly (brand new, kept under vacuum) and another recycled from previous PBF processes. The PBF machine used to produce the RAI samples was an EOS M290 (E.O.S. GmbH, Krailling, Germany) equipped with a Yb fibre laser of 400 W, with a scanning speed of up to 7.0 m/s and a focus diameter of 100 μm. All processing was made under inert Ar gas. After the production, the RAI samples were collected from the tray and stocked in a dry flask to avoid contamination. The control powder samples were collected from the sealed flask. The used powder samples were collected from the top of the powder bed (3D printer tray) after at least 20 impressions. After each impression, the remaining powder was transferred to the feed chamber; the machine was cleaned, and a new build plate was installed after each fabrication. Implant_C and implant_U were manufactured from controlled clean powder and used powder, respectively.

Kratos Axis Ultra X-ray Photoelectron Spectroscopy (Wharfside, Manchester, UK) was used to determine the chemical surface composition of the Ti-6Al-4V alloy samples. The parameters used included a vacuum base pressure of 2 × 10^−9^ torr, an X-ray gun emission set to 15 mA, and an X-ray gun anode HT set to 15 kV, which equates to a power setting of 225 W. The elements detected were observed using both survey and high-resolution spectra. Additional factors included a hybrid lens (magnetic and electrostatic), the use of a charged neutralizer during data acquisition, and an aperture set to 700 × 300 μm. A pass energy of 160 eV was used during the survey scan, while a pass energy of 20 eV was used during the high-resolution scan. Argon sputtering was used to create depth profiling. Data were acquired (*n* = 9) before etching with argon (0 nm) and after etching at depths of 10 nm and 100 nm (see the graphical abstract). Measurement spots were selected on different sample locations to detect the chemical composition’s consistency. Casa Software Ltd. was used to analyze the XPS results. The analysis was conducted after the XPS binding energy values were calibrated and the charge was corrected to that of uncharged carbon (CH—CH) at 285.0 eV. XPS high-resolution peak deconvolutions were performed using Shirley background removal for the peaks Ti2p, O1s, and C1s.

A JEOL JSM-7600TFE scanning electron microscope was used to image the surface structure and topography. Using the SEI detector, accelerating voltages for all samples were 10 kV, and magnifications were 50- and 200-micron scale.

## 3. Results

### 3.1. XPS Survey Analyses

The surface chemical composition (0 nm) results from XPS survey analyses are displayed in Table 1. At 0 nm, the amount of adventitious C was very high in all samples, especially on the implant surfaces. This fact may mask the amount of other major IIr elements: Ti, Al, and V. XPS Survey analyses also revealed the presence of trace elements, such as Na, Si, Zn, Ca, Cu, Mg, and P. Most of these elements were found mainly in the implants made with used powder.

**Figure 1:**
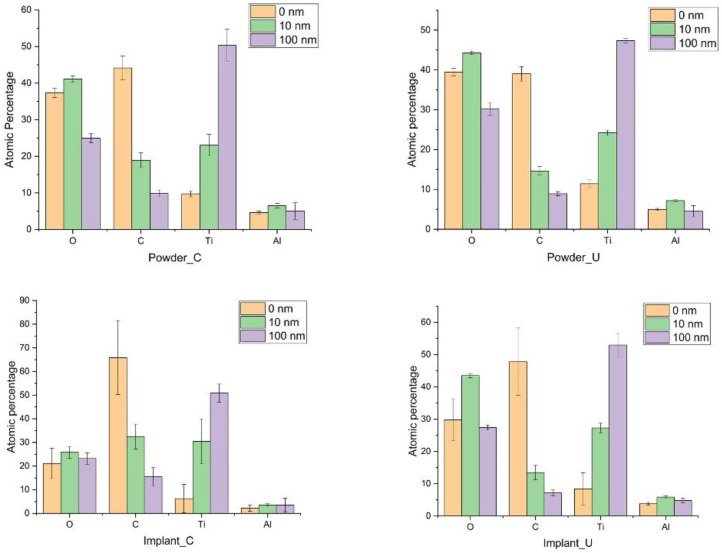
The atomic percentage of the major elements of the samples at three probed depths.

Figure 1 shows the atomic percentage of the major components (C, O, Ti, Al) according to the probed depth (0 nm, 10 nm, 100 nm). After the etching with Ar, the amount of C was reduced at 10 nm depth and more significantly at 100 nm. Implant_C presents the highest amount of carbon in all three depths. At the outmost layers, the presence of V was minimal (<1%), and it was omitted from the figures.

The amount of O in the three depths was almost the same between the control powder and the used powder but different from the implant samples. The powder oxidization result is consistent with the study by Cao et al. on recycled powders [35]. For example, at the Implant_C surface (0 nm), a small percentage of the O 1s peak was detected compared to Implant_U, 21.1 ± 6.4% and 29.8 ± 6.5%, respectively. However, at 10 nm depth, a significant difference in the oxygen concentration between Implant_C (25.8 ± 2.4%) and Implant_U (43.5 ± 0.6%) was observed (Figure 1). Higher oxygen content in the reused powder and the subsequent implant at the surface was expected and followed the literature data [20,36,37,38]. Oxygen is an α phase stabilizer, and its presence sometimes tends to increase the alloy’s mechanical properties. Nonetheless, our data showed visible oxygen increasing in the subsurface, at 10 nm deep, for Implant_U. This finding shows the increase in solubility of the oxygen in the subsurface. This phenomenon could be related to the action of the additional sources of oxygen, such as the accumulation of moisture in the reused powder and kept captive in the subsurface. Moreover, it could be an effect of the shielding gas. The shielding gas is applied to inhibit the side effects of the interaction of the melt pool vapour plume with the laser and can be a source of oxygen from helium impurities [36].

In the survey mode, the XPS analyses did not find a significant difference in the amount of Ti among all experimental conditions. However, minor variations for Al were also detected at 10 nm depth. Tang et al. observed that Al content decreases significantly from powder to printed samples [37].

**Figure 2:**
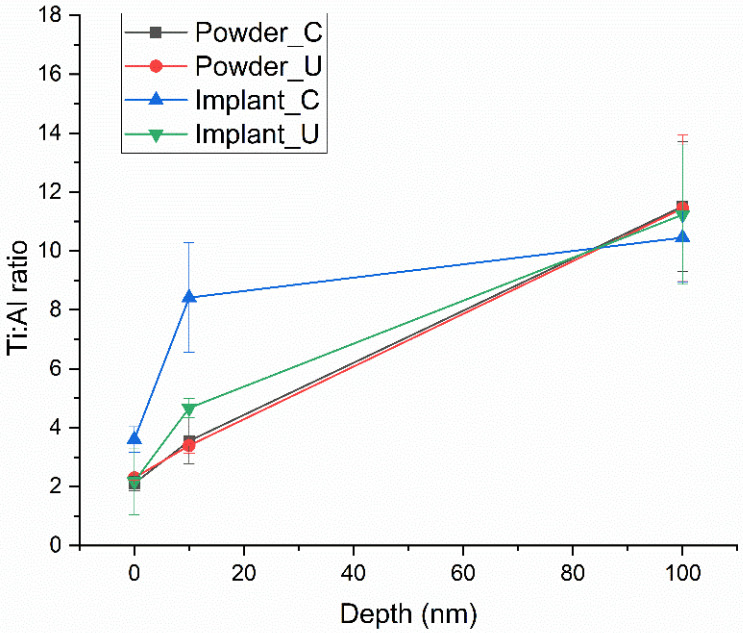
Ti/Al ratio average of the four experimental conditions, showing a significate difference at 10 nm for Implant_C.

The Ti/Al average ratio from the four experimental conditions is shown in Figure 2. Control and reused powder had the same ratio in all probed depths. However, Implant_C showed a decrease in Al content at 10 nm, followed by a less expressive difference from Implant_U. These findings confirm Tang et al.’s data.

### 3.2. XPS High-Resolution Analyses

**Figure 3:**
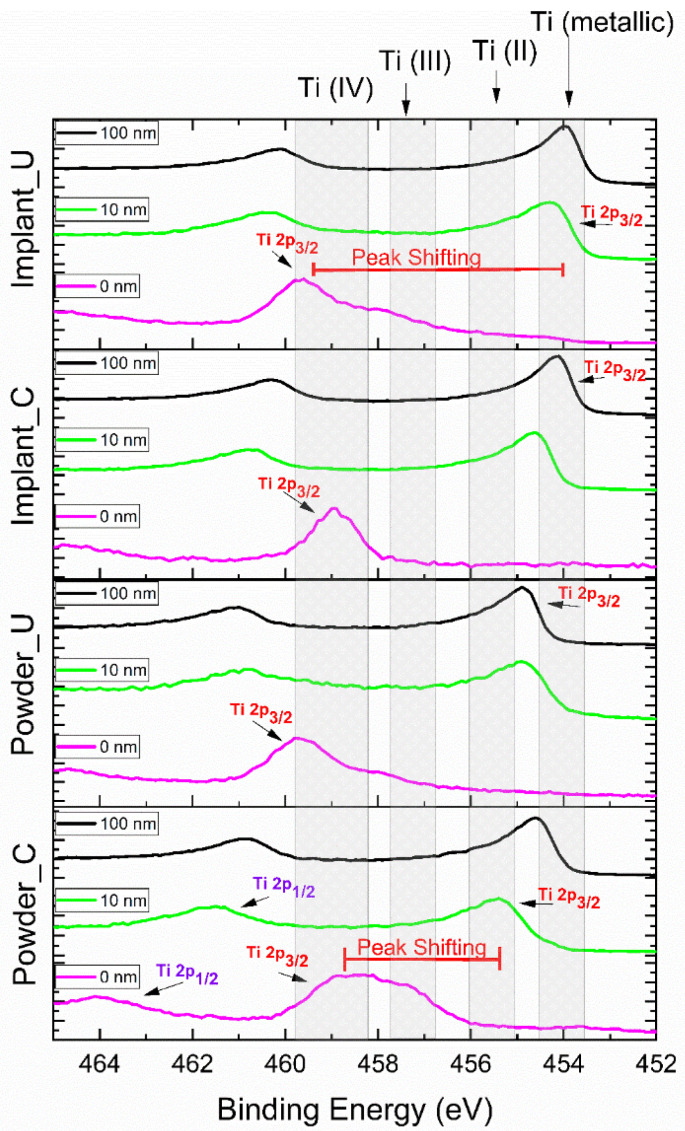
High-resolution XPS results for Ti 2p peak, according to the probed depth. Ti 2p is composed of two spin-orbit peaks: Ti 2p3/2 and Ti 2p1/2. The Ti 2p3/2 peak sifting from 0 nm to other experimental conditions was noticeable.

Table 2 shows the high-resolution XPS results for the atomic percentage of the Ti 2p peak contribution (%). The high-resolution XPS spectra for the Ti 2p peak are also presented in Figure 3. Unlike the XPS survey mode, the high-resolution spectra results provided a clearer picture of the oxidation states. Moreover, using peak deconvolution, it was possible to quantify these oxidation states; the percentages of the contributions can be seen in Table 2. As shown in Figure 3, Powder_C samples at 0 nm had the main contribution of the Ti 2p3/2 peak in the region of 458.8 eV (41.9%), indicating the presence of Ti (IV) or TiO_2_ [39,40,41,42]. Furthermore, Powder_C at 0 nm had a broad peak, indicating the presence of a high concentration of other oxidation states (20.9% of TiO and 37.2% of Ti_2_O_3_). On the other hand, the implant built from control powder, Implant_C at 0 nm, presented a high concentration (94.9%) in the TiO_2_ region. The samples from the used powder, Powder_U, and Implant_U presented the TiO_2_ peaks at high binding energy (489.7 eV) in comparison with the control ones. This finding confirms that recycling can contribute to a more oxidized outmost layer [36,43]. After the sputtering with Ar, all experimental conditions showed a peak shifting to low-binding energy regions. This shifting was less intense for Power_C at 10 nm and more intense for Implant_C at 100 nm. Powder_U at 10 nm and 100 nm was shown to have a high content of Ti (II) or TiO, and any percentage of TiO_2_ was noticed. At 454 eV, Ti (metallic) presence was detected in all samples at 100 nm depth, at 10 nm except for Powder_C, and in a small amount at 0 nm for Implant_U samples. This finding may correlate with the thickness of the passivation layer [39,41,43,44].

XPS analysis of the used powder surface showed a prominent peak for Ti^4+^ at the surface, representing the native oxide layer (TiO_2_, rutile) spontaneously formed on the titanium surface when in contact with air. After implant fabrication from both clean and reused powders, the oxide layer was detected on the implant’s surface by XPS. This finding indicates that the oxide layer on both Implant_C and Implant_U surfaces were stable and thick, which can be assumed from the absence of the metallic bulk state TiO contribution. A small amount (5.7%) of this state was presented on the surface of the Implant_U exclusively (Table 2).

The literature has reinforced the importance of Ti^4+^ in biomedical applications. TiO_2_-based surfaces are known for their ability to expedite and boost the capacity of protein absorption. In addition, the presence of TiO_2_ on the surface is critical for osteointegration as this oxide increases surface hydrophilicity. This high wettability causes biological effects such as attachment of fibroblasts, in vitro apatite formation, expression of osteogenic genes, and new bone formation. It also provides adhesion, proliferation, and osteogenic differentiation of MSCs cells [45,46]. Our findings have shown that the TiO_2_ is only present at the outmost layer (0 nm), except for Powder_C (10.9% at 10 nm), and confirm some literature data that show that the TiO_2_ film is about 1–6 nm [42,47,48,49].

**Figure 4:**
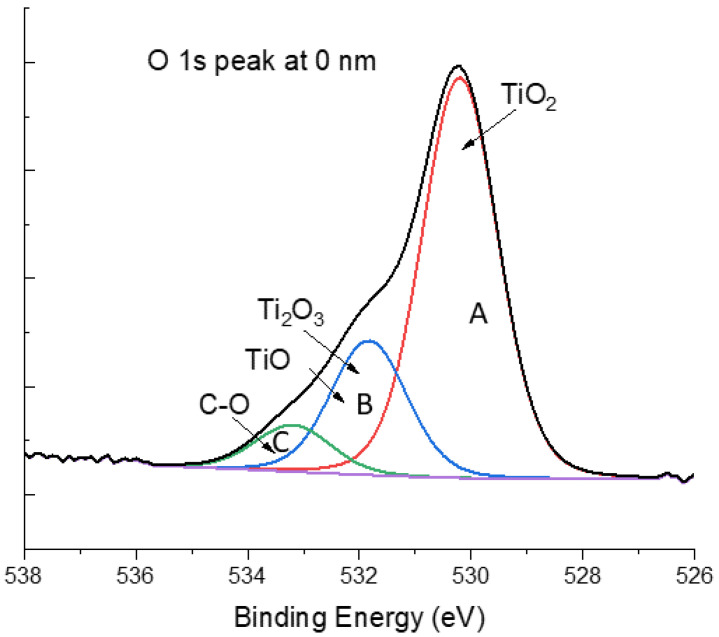
XPS high-resolution peak of oxygen peak O 1s at 0 nm for Powder_U samples. The three major contributions are O 1s_A, O 1s_B, and O 1s_C.

The XPS high-resolution oxygen peak, O 1s, was also analyzed for all experimental conditions. As shown in Figure 4, three major contributions were detected in all tested depths. The contribution O 1s_A at ≈530 eV was assigned to the main phase TiO_2_; the contribution O 1s_B at ≈532 eV can be attributed to two phases, Ti_2_O_3_ and TiO; and the contribution O 1s_C at ≈533 eV was related to the interaction between oxygen and organic elements such as carbon. All the assignments were according to XPS data from the National Institute of Standards and Technology [50]. However, the deconvolution of the high-resolution O 1s peak did not bring much more information due to the overlapping of the two phases Ti_2_O_3_ and TiO. Moreover, the presence of a high concentration of adventitious carbon on the surface of the samples, and its consequent oxidation, reduced the possibility of correlating this data with those in Table 2.

### 3.3. Scanning Electronic Microscopy

**Figure 5:**
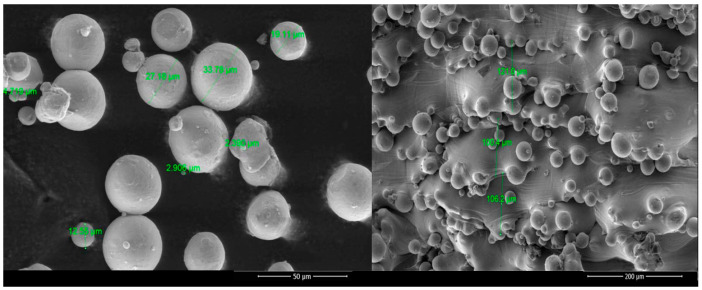
SEM images of the Ti6Al4V alloy powder and the surface of the Ti6Al4V implants.

SEM images are displayed in Figure 5 for the powder and the implant surfaces. The size of the alloy powder was between ≈25 and 35 µm, and after being fused by the laser, the implants presented residual powder partially melted at the surface. No topographic differences were noticed between the virgin and reused powder or between the different kinds of implants.

## 4. Discussion

In conventional dental implants, a surgical procedure drills a hole in the jawbone, and a screw-shaped implant is placed in direct contact with blood and bone cells. Custom-making R.A.I.s are planned to be used without a drilling step, using the extracted dental root’s hole. Titanium additively manufactured implants are suitable for immediate implantation due to the PBF processes’ ability to manufacture complex structures without additional tooling or post-processing operations [51]. Using the hole left from the extracted tooth is an advantage to RAI. However, it makes the surface composition of RAI even more critical because there will not be threads, as is the case in the conventional implant. It is expected that the bone cells start to form a new bone matrix at the implant surface, with little or no inflammation. At the nano level, the control of inflammation and the speed of bone formation are related to the absorption at the surface (0 nm) of biomolecules from the blood. In the first minutes of the implantation, there is clear evidence that the proteins and other biomolecules will be guided essentially by the chemical content of the implant’s outermost layer (0 nm) [27,52,53,54].

This background is essential to understanding the importance of this study. Our results indicated that root-analog dental implants from unspoiled and reprocessed powder have almost the same chemical composition at the surface (0 nm). However, the quality and the quantity of the oxide layer formed were different. For example, implants using the control powder had, on average, 94.9% of TiO_2_ at 0 nm, against 55.6% for those using recycling powder. The presence of TiO_2_ on the surface exhibits better osteointegration and reduces bacterial infection of Streptococcus sanguinis and Lactobacillus salivarius without jeopardizing implant efficiency [55,56,57].

Analyses of other presented depths have shown that the oxide layer may include all oxidation types (Ti^4+^, Ti^3+^, Ti^2+^). The presence of the metallic state of titanium is higher on deeper layers. The quality and quantity of titanium oxides are essential as they bond to living bone onto implants’ surface through a formed apatite layer. The apatite layer modulates the adhesion, migration, proliferation, differentiation, and consequent bone formation responsible for secondary stability [23,58]. Higher roughness of the PBF surface also contributes to a better coagulum stability condition, facilitating bone healing on the implant surface [59].

This pioneering study can provide a good contribution to future studies intending to improve RAI fabrication using the PBF technique. This process still has many variables. For instance, the powder bed temperature can sometimes be between 2000 and 3000 °C. The effect of such magnitude of temperature variation on reminiscent powder over time can produce an undesirable effect on the surface and compromise the optimal performance of the implant. A powerful surface technique such as XPS analyses allowed us to detect and quantify the atomic elements on the outmost layer of the fabricated implants. This study was able to detect the evaporation of Al at 10 nm depth, as previously recognized by Cao et al. and Tang et al. [34,35,36]. However, it is essential to point out the limitations of the XPS technique. The detection limit of XPS is 0.1–1% depending on the chemical element. Our research group has faced the effect of this limitation in our previous publications [60,61]. A complementary technique, such as time-of-flight secondary ion mass spectrometry, could bring light to detect traces elements and understand the role of the organic elements on the surface [62].

The results from in vitro studies involving RAI and cells seem to be promising. Adaptation of the bone to the micro-irregularities of the implant surface with developing osteocyte lacunae was shown by Mangano, F., et al. [63]. Maher, S., et al. (2021) noted that additively manufactured titanium implants have host cell attachment and bone mineralization and can potentially provide antibacterial protection [64]. The good condition of the peri-implant tissues was confirmed by the radiographic examination in many cases for the RAI implantation with a high survival rate within a year to three year follow-up [10,12,65,66].

Structural and functional connection between surrounding bone and implant surface is required to achieve successful osseointegration. Conventionally manufactured dental implants are made from titanium rods and require intensive surface treatment to achieve desired surface properties. RAI fabrication by AM allows us to have a porous structure with micro- and nano-geometry parameters for favorable biological response and ability to osseointegrate [67,68,69]. In addition, multiple studies have underlined the influence of surface roughness on the expression of osteogenic markers and modulating the cell behavior [70,71].

A porous RAI surface has multiple biological benefits. While RAI has enhanced fracture resistance due to the shape, similar to the root of the natural tooth, porosity permits the circulation of biological fluids and nutrients [19,66]. Porosity on the implant’s surface promotes bone ingrowth into pores and provides anchorage for biological fixation and a system that enables stress transfer and long-term stability.

The AM manufacturing process can be used to fabricate implants with a defined and well-controlled porosity to enhance bone attachment [67,72]. As suggested by other research group, the implant surface can be modified further to create micro-rough, nano-rough, and hydrophilic surfaces [67].

Unfortunately, manufacturing from reused powder will degrade the surface characteristics of the implant due to the changes of particle shape and less uniform layers [38]. Wettability and surface free energy measurements of the implant surface will be required to better understand the implant’s surface characteristics manufactured from reused powder. While the possibility of assessing the long-term success of RAI and promoting custom patient rehabilitation is well known, future studies should focus on the influence of the surface geometry of the RAI on surface oxidation. Moreover, questions regarding the impact of the increase in surface thermodynamics and the variations of the chemical composition at the nano level should not be ignored.

## 5. Conclusions

This study evaluated the composition of clean and reused titanium powder and manufactured implants at three different depths. Chemical composition for two groups of samples was assessed: (1) Unused powder and recycled powder, where we can see the peak of binding energy shifting with the depth increase. The kind of oxidation also changes with more oxide on the surface (especially TiO_2_) presented in the recycled powder composition. (2) RAI is manufactured from pure and reused powder accordingly.

XPS observations revealed the presence of different kinds of titanium oxidization with the high presence of TiO_2_ on the surface of implants and powder. This oxide is known as being favourable for osteointegration, antibacterial resistance, and corrosion resistance. Furthermore, clean and recycled powder implants have demonstrated a thick layer of this oxide. These results indicate that even with the degradation of powder properties through the PBF process, the surface oxide layer has a sufficient presence of TiO_2_. The above results show that Ti6Al4V powder could be used in up to 20 cycles for RAI manufacturing. This study provides insight into finding a reasonable balance between desired implant properties and reduced manufacturing costs.

## Figures and Tables

**Table 1:** Atomic percentage and standard deviation of the tested samples at the surface (0 nm).

	Control Powder		Used Powder		Implant_C		Implant_U	
Elements	%	SD	%	SD	%	SD	%	SD
O	37.4	1.3	39.4	1.0	21.1	6.4	29.8	6.5
C	44.1	3.3	39.0	1.8	65.9	15.6	47.8	10.4
N	0.8	0.3	0.8	0.3	0.3	0.3	0.7	0.6
Na	0.6	0.4	0.7	0.2	1.1	1.6	0.1	0.1
Ti	9.7	0.8	11.4	1.0	6.2	6.0	8.4	5.0
Si	2.7	0.8	2.7	0.7	1.9	0.7	0.3	0.6
Al	4.6	0.4	5.0	0.3	1.5	1.6	3.8	0.4
Zn	0.0	0.0	0.0	0.0	0.0	0.0	0.6	0.1
Ca	0.0	0.0	0.0	0.0	1.4	0.2	1.3	0.1
V	0.0	0.0	0.0	0.0	0.1	0.1	0.1	0.1
Cu	0.0	0.0	0.0	0.0	0.0	0.0	0.2	0.1
Mg	0.0	0.0	0.0	0.0	0.0	0.0	6.3	1.4
P	0.0	0.0	0.0	0.0	0.0	0.0	0.2	0.4

**Table 2:** High-resolution XPS results: atomic percentage of the Ti 2p peak contribution (%).

		Powder_C			Powder_U			Implant_C			Implant_U		
Peak Position (eV)	Compound	0 nm	10 nm	100 nm	0 nm	10 nm	100 nm	0 nm	10 nm	100 nm	0 nm	10 nm	100 nm
454.0 (±0.4)	Ti (metallic)	-	-	62		8.6	4.5	-	31.2	60.7	5.7	58.9	60.4
455.8 (±0.6)	Ti (II)-TiO	20.9	63.7	25.7		65.6	64.5	-	44.8	27.9	11.9	28.5	26.1
457.4 (±0.5)	Ti (III)-Ti_2_O_3_	37.2	25.4	12.3	43.7	25.8	30	5.1	24	11.4	26.8	12.6	13.6
458.8 (±0.4)	Ti (IV)-TiO_2_	41.9	10.9	-	56.3			94.9	-	-	55.6	-	-

## Data Availability

The data presented in this study are available on request.
